# Supplementary material for: MicroRNA156 and its targeted SPL genes interact with the photoperiod, vernalization, and gibberellin pathways to regulate wheat heading time
Source: Plant J. 2026 Jan 11;125(1):e70656. doi: 10.1111/tpj.70656 (PMC12790879; doi:10.1111/tpj.70656)

Supplementary Figures

Figure S1. miR156 binding sites in nine wheat *SPL* genes and their expression profiles.

(**a**) miR156 binding sites of wheat *SPL* genes. Green letters represent natural polymorphisms within the conserved miR156 binding site.

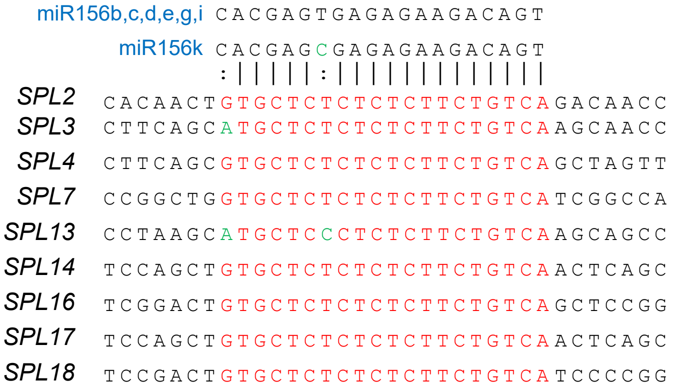

(**b**) Expression profiles of the A, B and D homeologs of the nine wheat *SPL* genes with miR156 binding sites based on ExpVIP (http://www.wheat-expression.com/, $\log_2$ TPM). The three rows in root and leaf/shoot represent samples collected at seedling, vegetative and reproductive stages, respectively. This graph is based on four RNA-seq studies in hexaploid wheat selected from ExpVIP: Developmental time courses in CS and Azhurnaya, CS leaves and roots from 7 leaf stages and CS seedlings (leaf and roots) and developing spikes.

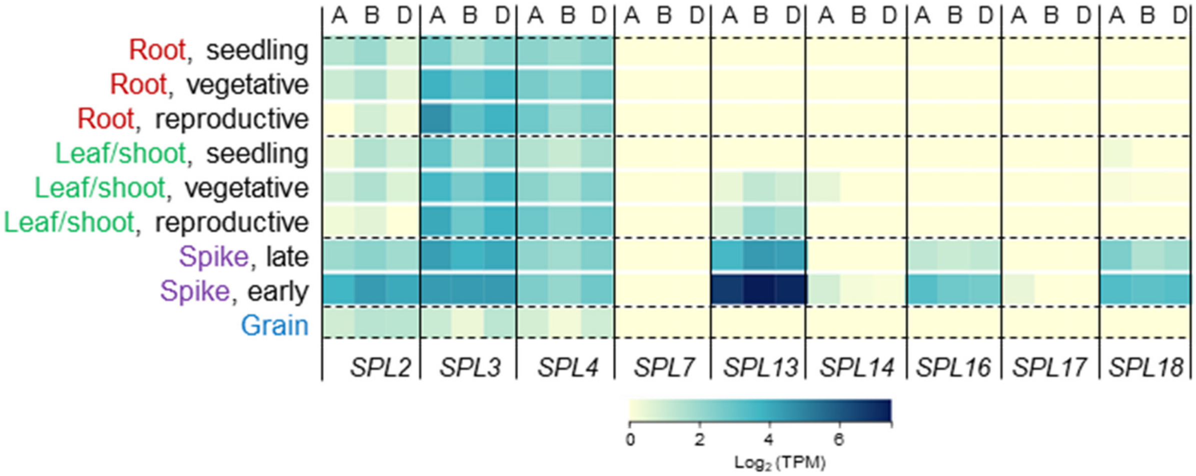

Figure S1. Continuation.

(**c**) Single molecule fluorescent in situ hybridization (smFISH) of miR156 regulated SPLs at the transition of the shoot apical meristem (SAM) into an inflorescence meristem (IM) in tetraploid wheat Kronos. Data was obtained from X. Xu et al. (2025). Genome Biology 26:352. https://doi.org/10.1186/s13059-025-03811-3

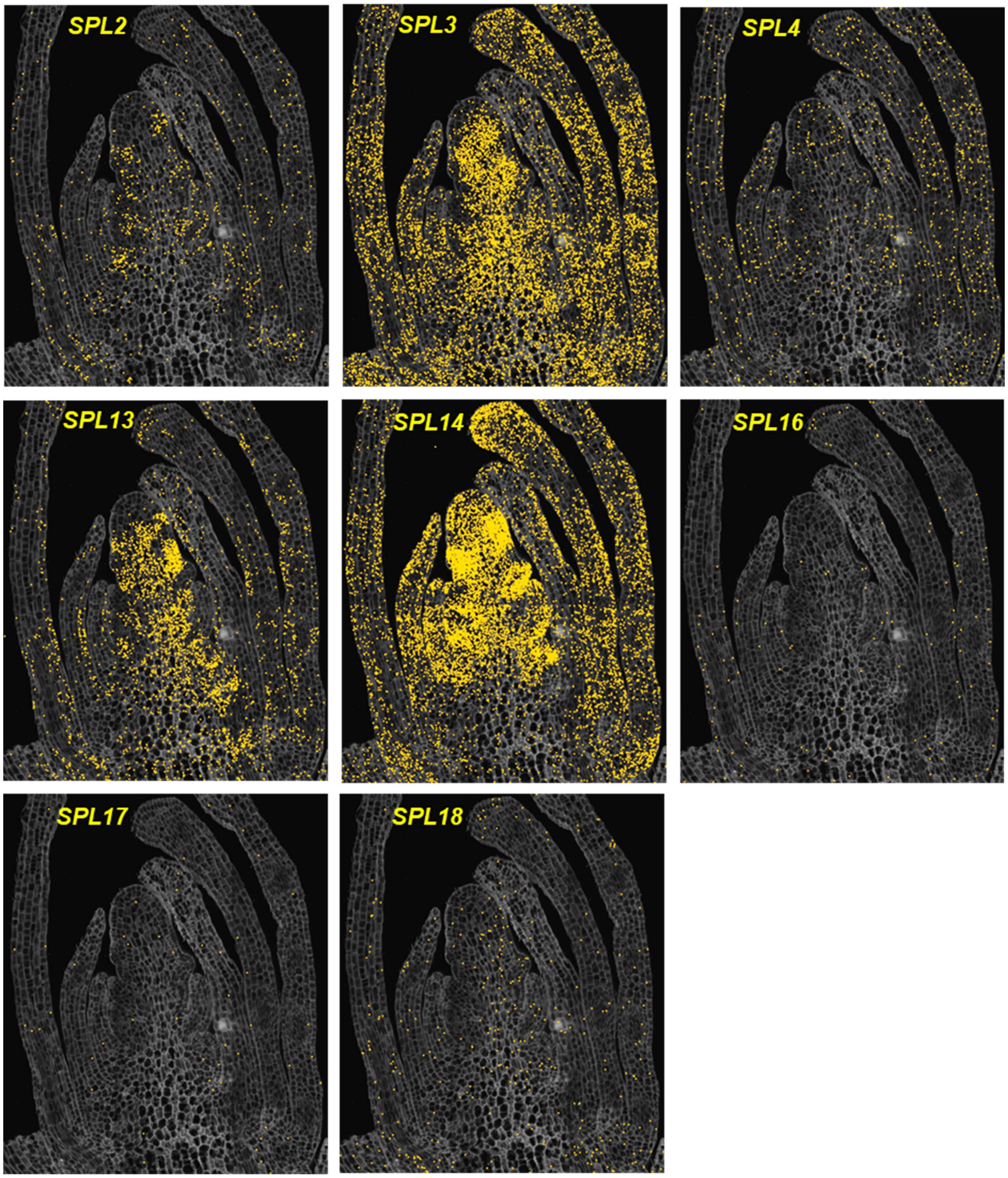

Figure S2. Alignment of SBP domains for Arabidopsis, rice, and wheat SPL proteins.

```
           ....:...10....:...20....:...30....:...40....:...50....:...60....:...70....:...80....
TaSPL-A18  CAVDGCKADLSKC-RDYHRRHKVCEAHSKTPLVV-VAGREMRFCQQCS-------RFHLLAEFDEAKRSCRKRLDGHNRRRRK-
OsSPL16    CAVDGCKEDLSKC-RDYHRRHKVCEAHSKTPLVV-VSGREMRFCQQCS-------RFHLLQEFDEAKRSCRKRLDGHNRRRRK-
OsSPL18    CAVDGCKADLSKH-RDYHRRHKVCEPHSKTPVVV-VSGREMRFCQQCS-------RFHLLGEFDEAKRSCRKRLDGHNRRRRK-
TaSPL-A16  CAVDGCRADLSRC-RDYHRRHKVCEAHSKTPVVT-VAGREMRFCQQCS-------RFHLLTEFDETKRSCRKRLDGHNRRRRK-
TaSPL-A2   CSVEGCTADLSRC-REYHRRHKVCEAHSKTPVVA-VAGQQQRFCQQCS-------RFHLLGEFDEVKRSCRKRLDGHNRRRRK-
OsSPL2     CSVEGCAADLSKCVRDYHRRHKVCEAHSKTAVVT-VAGQQQRFCQQCS-------RFHLLGEFDEEKRSCRKRLDGHNKRRRK-
OsSPL19    CSVDGCRSDLSRC-RDYHRRHKVCEAHAKTPVVV-VAGQEQRFCQQCS-------RFHNLAEFDDGKKSCRKRLDGHNRRRRK-
AtSPL13    CLVDGCDSDFSNC-REYHKRHKVCDVHSKTPVVT-INGHKQRFCQQCS-------RFHALEEFDEGKRSCRKRLDGHNRRRRK-
TaSPL-A1   CQVDGCHADLGDD-RDYHRRHKVCEPHTKSTLVR-IRNIEHRFCQQCS-------RFHLVQEFDEGKKSCRSRLATHNRRRRK-
OsSPL1     CQVDGCTVNLSSA-RDYNKRHKVCEVHTKSGVVR-IKNVEHRFCQQCS-------RFHFLQEFDEGKKSCRSRLAQHNRRRRK-
TaSPL-A6   CQVEGCCADLSAA-KDYHRRHKVCEMHAKANTAV-VGNTVQRFCQQCS-------RFHLLQEFDEGKRSCRRRLAGHNKRRRK-
OsSPL6     CQVEGCTADLTGV-RDYHRRHKVCEMHAKATTAV-VGNTVQRFCQQCS-------RFHPLQEFDEGKRSCRRRLAGHNRRRRK-
AtSPL1     CQVENCEADLSKV-KDYHRRHKVCEMHSKATSAT-VGGILQRFCQQCS-------RFHLLQEFDEGKRSCRRRLAGHNKRRRK-
AtSPL12    CQVDNCGADLSKV-KDYHRRHKVCEIHSKATTAL-VGGIMQRFCQQCS-------RFHVLEEFDEGKRSCRRRLAGHNKRRRK-
AtSPL14    CQVDNCTEDLSHA-KDYHRRHKVCEVHSKATKAL-VGKQMQRFCQQCS-------RFHLLSEFDEGKRSCRRRLAGHNRRRR--
TaSPL-A15  CQVDDCRADLTSA-KDYHRRHKVCEIHSKTTKAV-VGNQMQRFCQQCS-------RFHPLSEFDEGKRSCRRRLAGHNRRRRK-
OsSPL15    CQVDDCRADLTNA-KDYHRRHKVCEIHGKTTKAL-VGNQMQRFCQQCS-------RFHPLSEFDEGKRSCRRRLAGHNRRRRK-
TaSPL-A8   CQAEGCKADLSGA-KRYHRRHKVCEHHSKAPVVVTAGGLHQRFCQQCS-------RFHLLDEFDDAKKSCRKRLADHNRRRRK-
OsSPL8     CQAEGCKADLSSA-KRYHRRHKVCEHHSKAPVVVTAGGLHQRFCQQCS-------RFHLLDEFDDAKKSCRKRLADHNRRRRK-
AtSPL8     CQAEGCNADLSHA-KHYHRRHKVCEFHSKASTVV-AAGLSQRFCQQCS-------RFHLLSEFDNGKRSCRKRLADHNRRRRK-
TaSPL-A10  CQVQDCKADLSGA-KHYHRRHKVCEYHAKAALVS-AAGKQQRFCQQCS-------RFHVLTEFDEAKRSCRRRLAEHNRRRRK-
TaSPL-A20  CQVEDCKADLSGA-KHYHRRHKVCEYHAKASLVA-AAGKQQRFCQQCS-------RFHVLTEFDEAKRSCRRRLAEHNRRRRK-
TaSPL-A23  CQVEYCKADLSGA-KHYHRRHKVCEYHAKAALVS-AAGKQQRFCQQCS-------RFHVITEFDQTKRSCRKRLAEHNRRRRK-
TaSPL-A5   CQAEGCKADLSAA-KHYHRRHKVCEYHAKATTVA-ASGKQQRFCQQCS-------RFHVLAEFDEAKRSCRKRLTEHNRRRRK-
OsSPL5     CQAEGCKADLSAA-KHYHRRHKVCDFHAKAAAVL-AAGKQQRFCQQCS-------RFHVLAEFDEAKRSCRKRLTEHNRRRRK-
TaSPL-A22  CQAEGCKADLSGA-KHYHRRHKVCEYHAKASLVA-AAGKQQRFCQQCS-------RFHVLTEFDEAKRSCRKRLAEHNRRRRK-
OsSPL10    CQAEGCKADLSGA-KHYHRRHKVCEYHAKASVVA-ASGKQQRFCQQCS-------RFHVLTEFDEAKRSCRKRLAEHNRRRRK-
TaSPL-A13  CQAERCGADLSEA-KRYHRRHKVCEAHAKAAVVV-VAGLRQRFCQQCS-------RFHELLEFDDQKRSCRRRLAGHNERRRK-
OsSPL13    CQVERCGVDLSEA-GRYNRRHKVCQTHSKEPVVL-VAGLRQRFCQQCS-------RFHELTEFDDAKRSCRRRLAGHNERRRK-
TaSPL-A14  CQVEGCGVDLSGG-KTYYCRHKVCLEHSKAPLVV-VAGIEQRFCQQCS-------RFHQLPEFDQGKRSCRRRLAGHNERRRK-
OsSPL14    CQVEGCGADLSGI-KNYYCRHKVCFMHSKAPRVV-VAGLEQRFCQQCS-------RFHLLPEFDQGKRSCRRRLAGHNERRRR-
TaSPL-A17  CQVEGCGVDLSGA-KQYHCRHKVCSMHTKEPRVV-VAGLEQRFCQQCS-------RFHQLPEFDQGKRSCRRRLAGHNERRRK-
OsSPL17    CQVEGCGVDLSGV-KPYYCRHKVCYMHAKEPIVV-VAGLEQRFCQQCSRCSVHMVRFHQLPEFDQEKKSCRRRLAGHNERRRK-
AtSPL9     CQVEGCGMDLTNA-KGYYSRHRVCGVHSKTPKVT-VAGIEQRFCQQCS-------RFHQLPEFDLEKRSCRRRLAGHNERRRK-
AtSPL15    CQVEGCRMDLSNV-KAYYSRHKVCCIHSKSSKVI-VSGLHQRFCQQCS-------RFHQLSEFDLEKRSCRRRLACHNERRRK-
AtSPL6     CQVYGCSKDLSSS-KDYHKRHRVCEAHSKTSVVI-VNGLEQRFCQQCS-------RFHFLSEFDDGKRSCRRRLAGHNERRRK-
TaSPL-A7   CQVEGCHMALAGA-KEYHRRHKVCEAHSKAPRVI-VHGAEQRFCQQCS-------RFHAMAEFDDAKRSCRRRLAGHNERRRK-
OsSPL7     CQVEGCDITLQGV-KEYHRRHKVCEVHAKAPRVV-VHGTEQRFCQQCS-------RFHVLAEFDDAKKSCRRRLAGHNERRRR-
TaSPL-A3   CQVEGCKADLSTV-KDYHRKHRVCELHAKAPKVV-VAGLERRFCQQCS-------RFHALAEFDQKKRSCRRRLNDHNSRRRK-
TaSPL-A4   CQVEGCGVELRDA-KEYHRKHRVCEAHTKFPRVV-VAGQERRFCQQCS-------RFHALSEFDQKKRSCRRRLSDHNARRRK-
OsSPL4     CQVEGCGVELVGV-KDYHRKHRVCEAHSKFPRVV-VAGQERRFCQQCS-------RFHALSEFDQKKRSCRRRLYDHNARRRK-
OsSPL11    CQVEGCGLELGGY-KEYYRKHRVCEPHTKCLRVV-VAGQDRRFCQQCS-------RFHAPSEFDQEKRSCRRRLSDHNARRRK-
OsSPL3     CQVEGCNVDLSSA-KPYHRKHRVCEPHSKTLKVI-VAGLERRFCQQCS-------RFHGLAEFDQKKRSCRRRLHDHNARRRK-
OsSPL12    CQVEGCKVDLSSA-REYHRKHKVCEAHSKAPKVI-VSGLERRFCQQCS-------RFHGLAEFDQKKKSCRRRLSDHNARRRK-
AtSPL2     CQVEGCNLDLSSA-KDYHRKHRICENHSKFPKVV-VSGVERRFCQQCS-------RFHCLSEFDEKKRSCRRRLSDHNARRRK-
AtSPL10    CQIDGCELDLSSS-KDYHRKHRVCETHSKCPKVV-VSGLERRFCQQCS-------RFHAVSEFDEKKRSCRKRLSHHNARRRK-
AtSPL11    CQIDGCELDLSSA-KGYHRKHKVCEKHSKCPKVS-VSGLERRFCQQCS-------RFHAVSEFDEKKRSCRKRLSHHNARRRK-
AtSPL3     CQVESCTADMSKA-KQYHKRHKVCQFHAKAPHVR-ISGLHQRFCQQCS-------RFHALSEFDEAKRSCRRRLAGHNERRRK-
ATSPL4     CQVDRCTADMKEA-KLYHRRHKVCEVHAKASSVF-LSGLNQRFCQQCS-------RFHDLQEFDEAKRSCRRRLAGHNERRRK-
AtSPL5     CQVDRCTVNLTEA-KQYYRRHRVCEVHAKASAAT-VAGVRQRFCQQCS-------RFHELPEFDEAKRSCRRRLAGHNERRRK-
TaSPL-A9   CQVPGCEADIREL-KGYHKRHRVCLRCAHSSAVM-IDGVQKRYCQQCG-------KFHILLDFDEDKRSCRRKLERHNKRRRRK
OsSPL9     CQVPGCEADIREL-KGYHRRHRVCLRCAHAAAVM-LDGVQKRYCQQCG-------KFHILLDFDEDKRSCRRKLERHNKRRRRK
AtSPL7     CQVPDCEADISEL-KGYHKRHRVCLRCATASFVV-LDGENKRYCQQCG-------KFHLLPDFDEGKRSCRRKLERHNNRRKR-
```

Figure S3. Phylogenetic tree of SPL proteins based on SBP alignment.

Phylogenetic tree based on SBP alignment (Figure S2, Method S1) using the Maximum Likelihood method and Le_Gascuel_2008 model (Le and Gascuel, 2008).

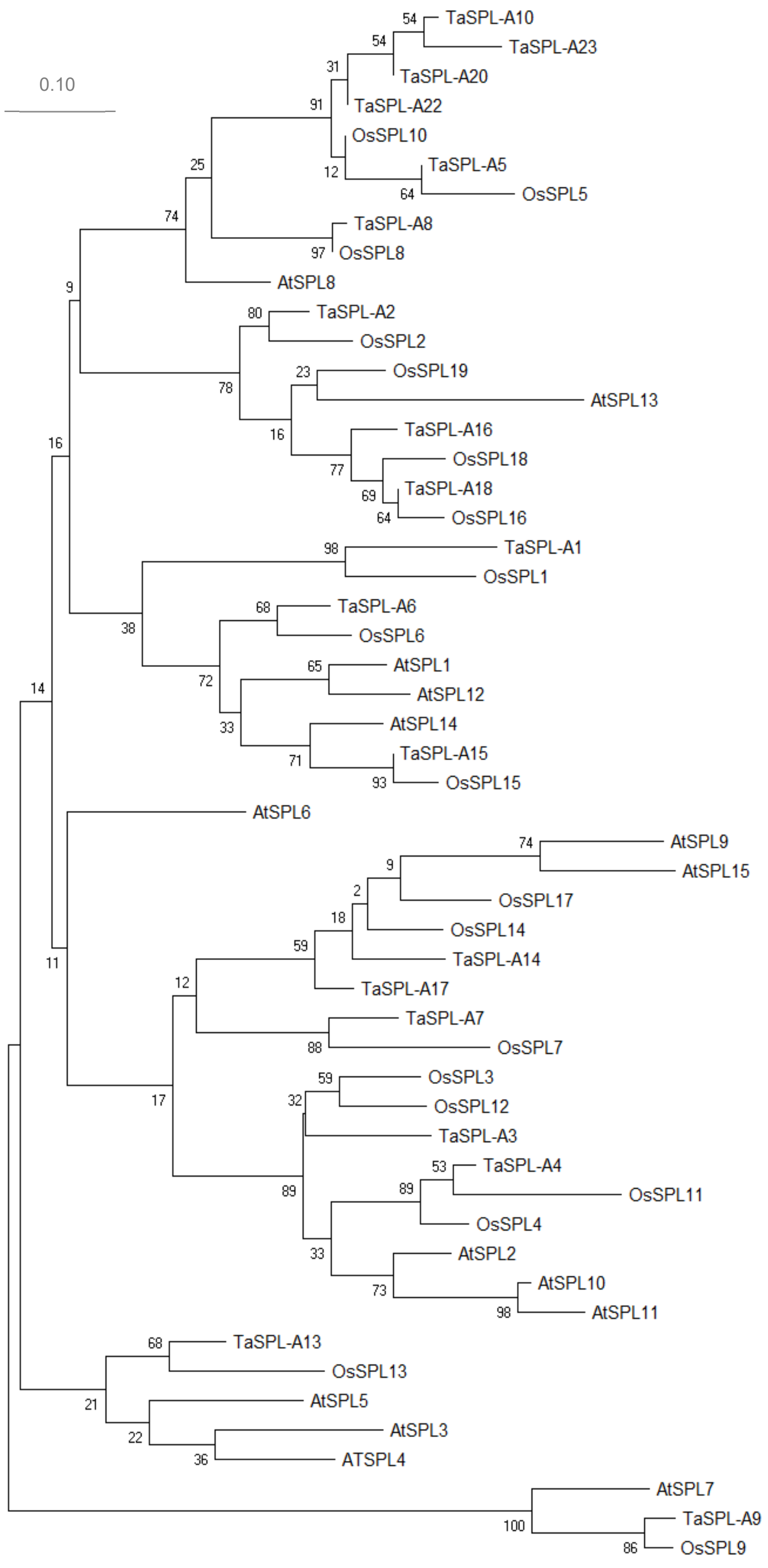

The percentage of trees in which the associated taxa clustered together is shown next to the branches. Initial tree(s) for the heuristic search were obtained the Neighbor-Join and BioNJ algorithms to a matrix of pairwise distances and then selecting the topology with superior log likelihood value. The tree is drawn to scale, with branch lengths measured in the number of substitutions per site. This analysis involved 53 amino acid sequences in the SBP domain (Figure S2). Evolutionary analyses were conducted in MEGA X (Kumar *et al.*, 2009).

References

1. Le S.Q. and Gascuel O. (**2008**). An improved general amino acid replacement matrix. *Mol Biol Evol* **25(7)**:1307-1320.

2. Kumar S., Stecher G., Li M., Knyaz C., and Tamura K. (**2018**). MEGA X: molecular evolutionary genetics analysis across computing platforms. *Molecular Biology and Evolution* **35**:1547-1549.

Figure S4. Regulation of *SPL3*, *SPL4*, *SPL13*, and *SPL2* expression by miR156.

(a-d) Boxplots showing expression of *SPL3*, *SPL4*, *SPL13*, and *SPL2* in leaves 1, 5 and 9 (n= 4). **(e-h)** Effect of overexpression of miR156 on *SPL3*, *SPL4*, *SPL13*, and *SPL2* expression (n= 6). Overexpression of miR156 occurs only when the pOp::miR156 and the Ubi::LhG4 are combined by crossing. **(i-l)** Effect of MIM156 on the transcript levels of *SPL3*, *SPL4*, *SPL13* and *SPL2* in five independent transgenic events. **(a-h)** Different letters indicate significant differences in Tukey tests ($P < 0.05$). Transcript levels are expressed relative to *ACTIN* and were calculated using the ΔCt method. **(i-l)** Dunnett tests between transgenics and wild-type, n= 4. ns= not significant, * = $P < 0.05$, ** = $P < 0.01$, *** = $P < 0.001$. Raw data and statistical analyses are available in Data S3.

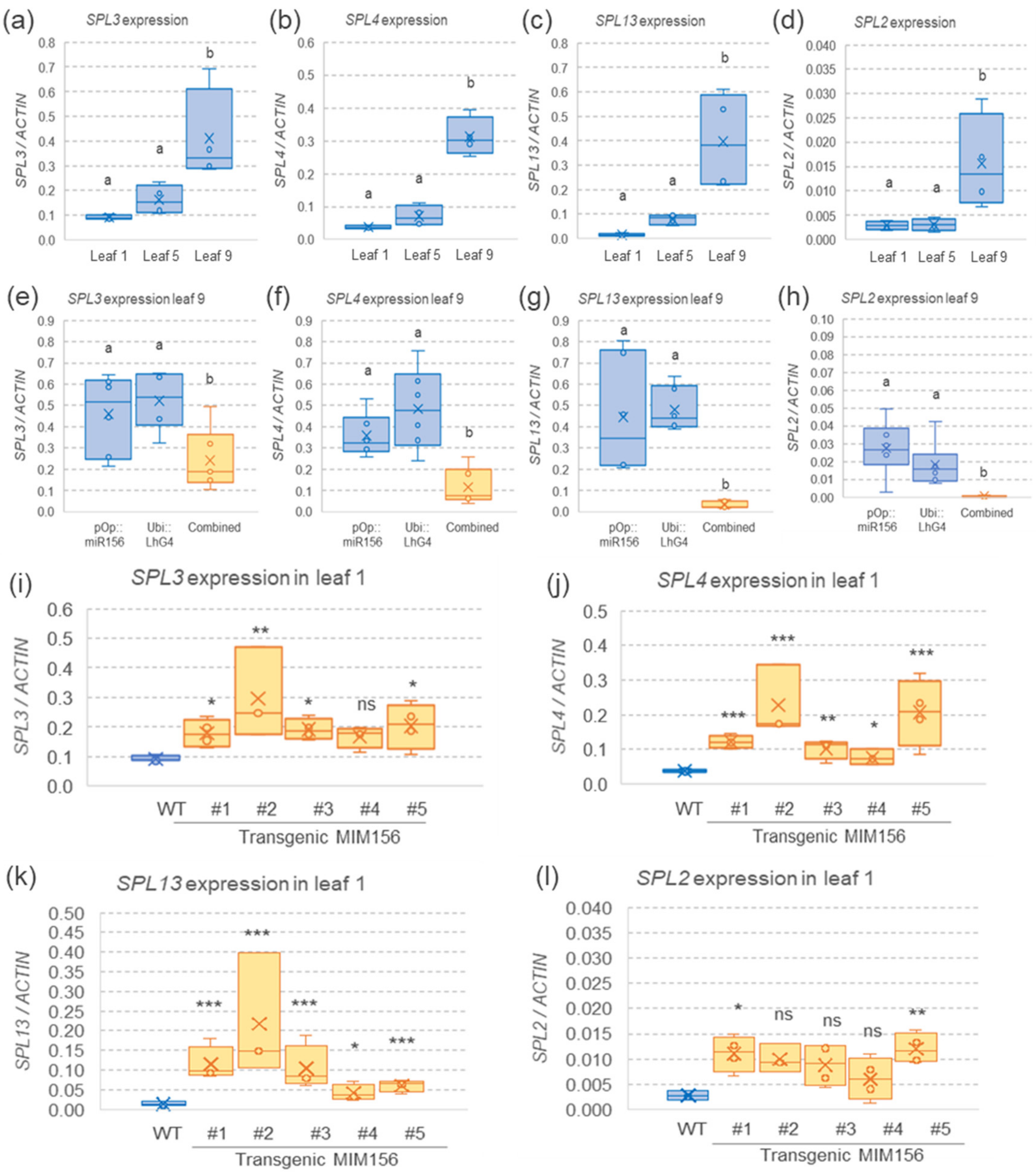

Figure S5. Expression of wild-type and resistant alleles *rSPL3*, *rSPL4*, and *rSPL13*.

(a) *rSPL-A3*, **(b)** *rSPL-B3*, **(c)** *rSPL-A4*, **(d)** *rSPL-A13* and **(e)** *rSPL-B13.* Transcript levels are expressed relative to *ACTIN* and were calculated using the ΔCt method. Probability values correspond to *t*-tests between resistant and wild-type alleles. ns= not significant, *= $P< 0.05$, **= $P< 0.01$, ***= $P< 0.001$. n= 6 plants per genotype (except for *rSPL-A4*, n=4). Raw data and statistical analyses are available in Data S3.

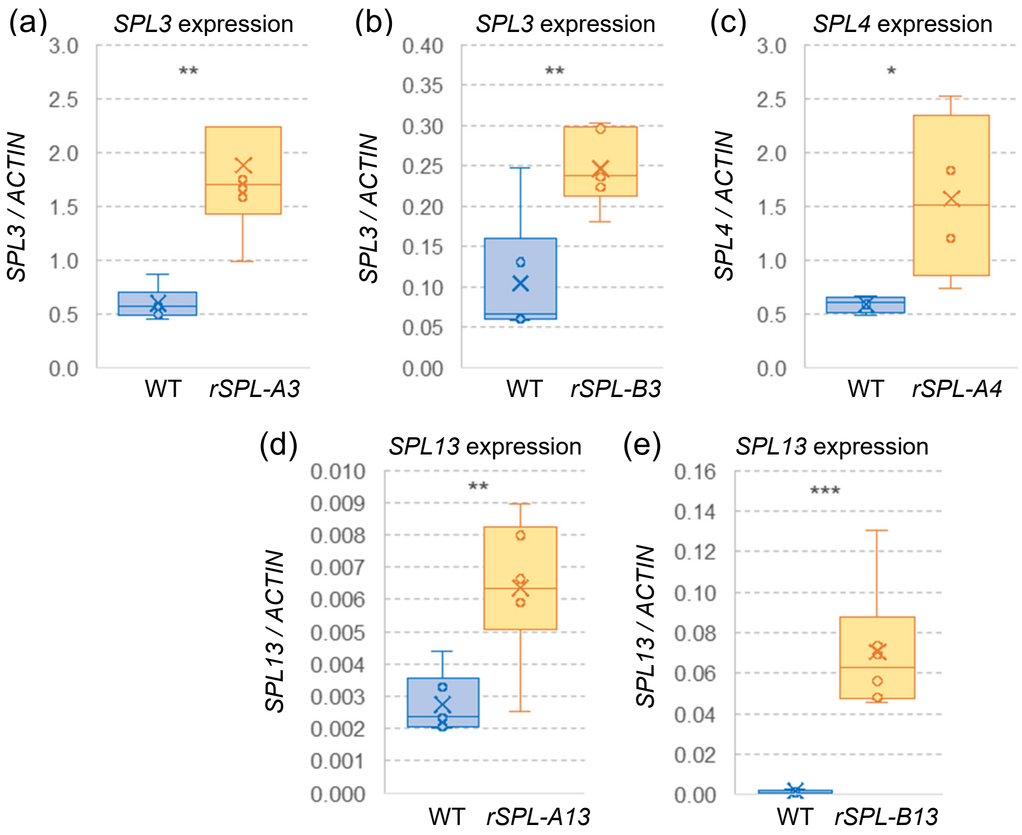

Figure S6. Pictures of wild-type, *rSPL3*, *rSPL4*, and *rSPL13* single mutants at heading time.

(**a**) *rSPL-A3*. (**b**) *rSPL-B3*. (**c**) *rSPL-A4*. (**d**) *rSPL-A13*. (**e**) *rSPL-B13*. Pictures were taken at the time of spike emergence in the mutant lines. Scale bar is 5 cm. Arrows at the top of the main tillers show emerging spikes in the *rSPL* mutants but not in their respective wild-type sister lines.

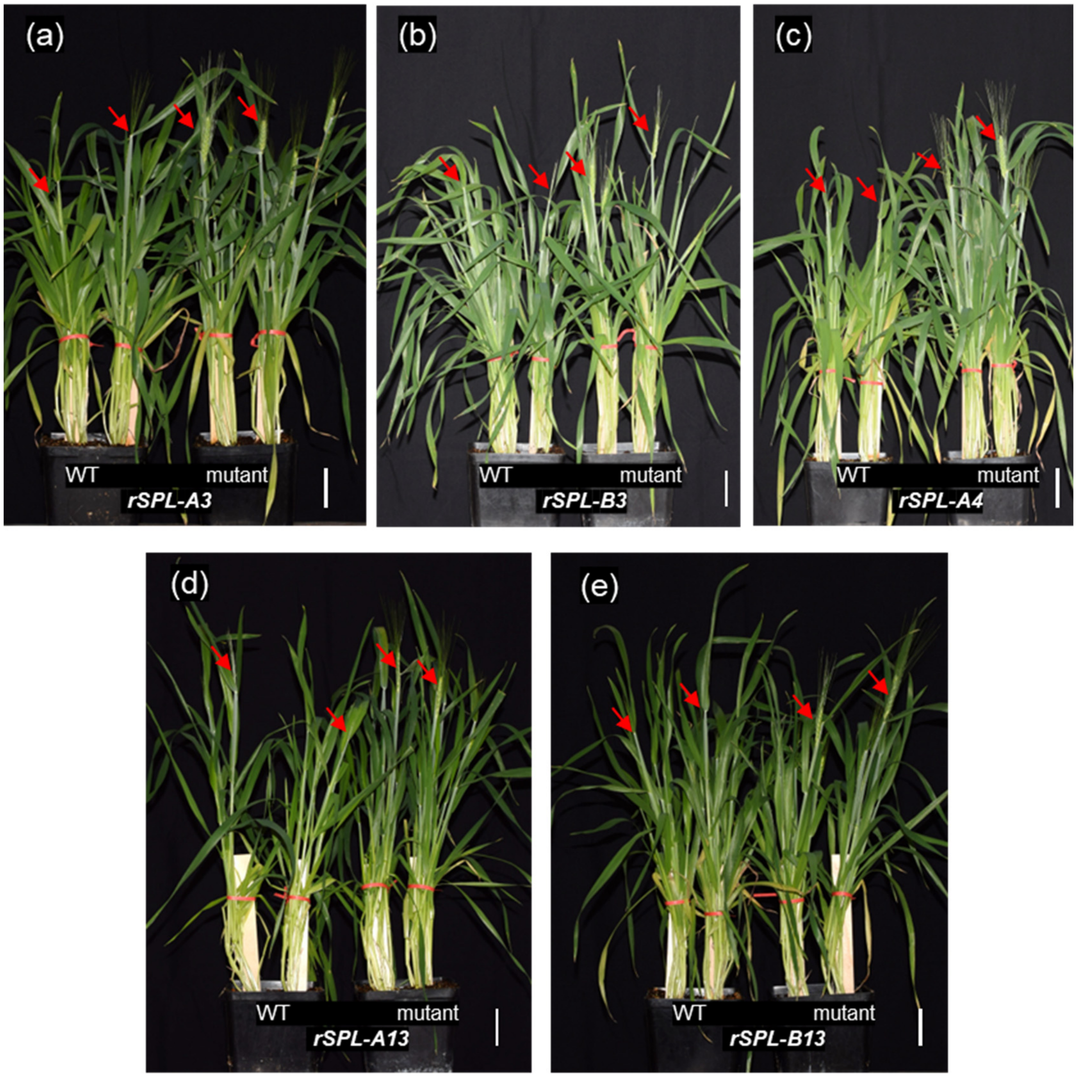

Figure S7. Effect of *rSPL3*, *rSPL4*, and *rSPL13* on leaf number.

(a) Leaf number of WT (n= 10) and r*SPL-A3* (n= 5). **(b)** Leaf number of WT (n= 14) and *rSPL-B3* (n= 9). **(c)** Leaf number of WT (n= 7) and *rSPL-B13* (n= 8). **(d)** Leaf number of single *rSPL-A4* and *rSPL-A13* and combined mutants compared with wild-type (n= 12). **(e)** Leaf number of triple resistant mutant *rSPL-B3/A4/A13* (n= 15) compared with WT (n= 14). ns = not significant, *= $P < 0.05$, **= $P < 0.01$, ***= $P < 0.001$. P values are from non-parametric Kruskal-Wallis test *vs.* wild-type. Non-parametric tests were used due to the lack of normality. Raw data and statistical analyses are available in Data S5.

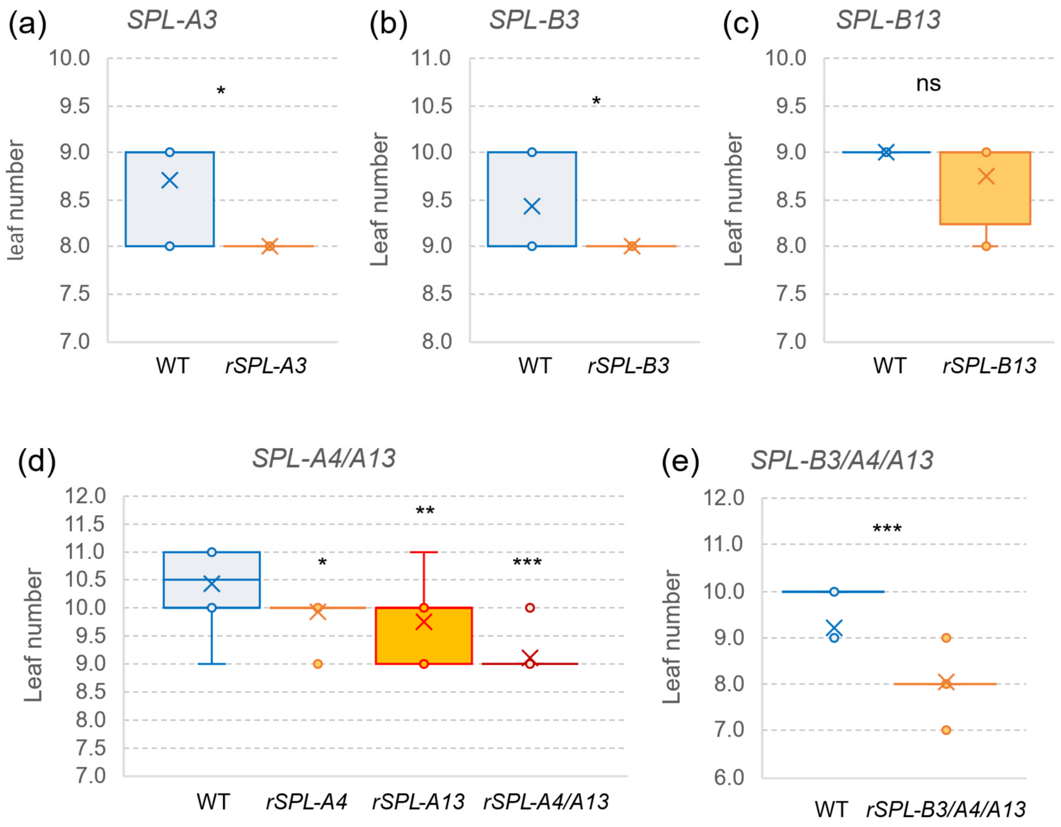

Figure S8. Effect of *rSP3*, *rSPL4*, and *rSPL13* alleles on heading time under LD and SD.

Interaction graphs for heading time of plants carrying different *SPL* resistant allele combinations grown under LD (16h light and 8 h darkness) and SD (8h light and 16 h darkness) conditions. **(a)** *rSPL-A4*, experiment 1. **(b)** *rSPL-A4*, experiment 2. **(c)** *rSPL-A13*. **(d)** *rSPL-B3*. **(e)** combined *rSPL-A4* / *rSPL-A13*. **(f)** combined *rSPL-B3* / *rSPL-A4* / *rSPL-A13*. Raw data and statistical analyses are available in Data S7.

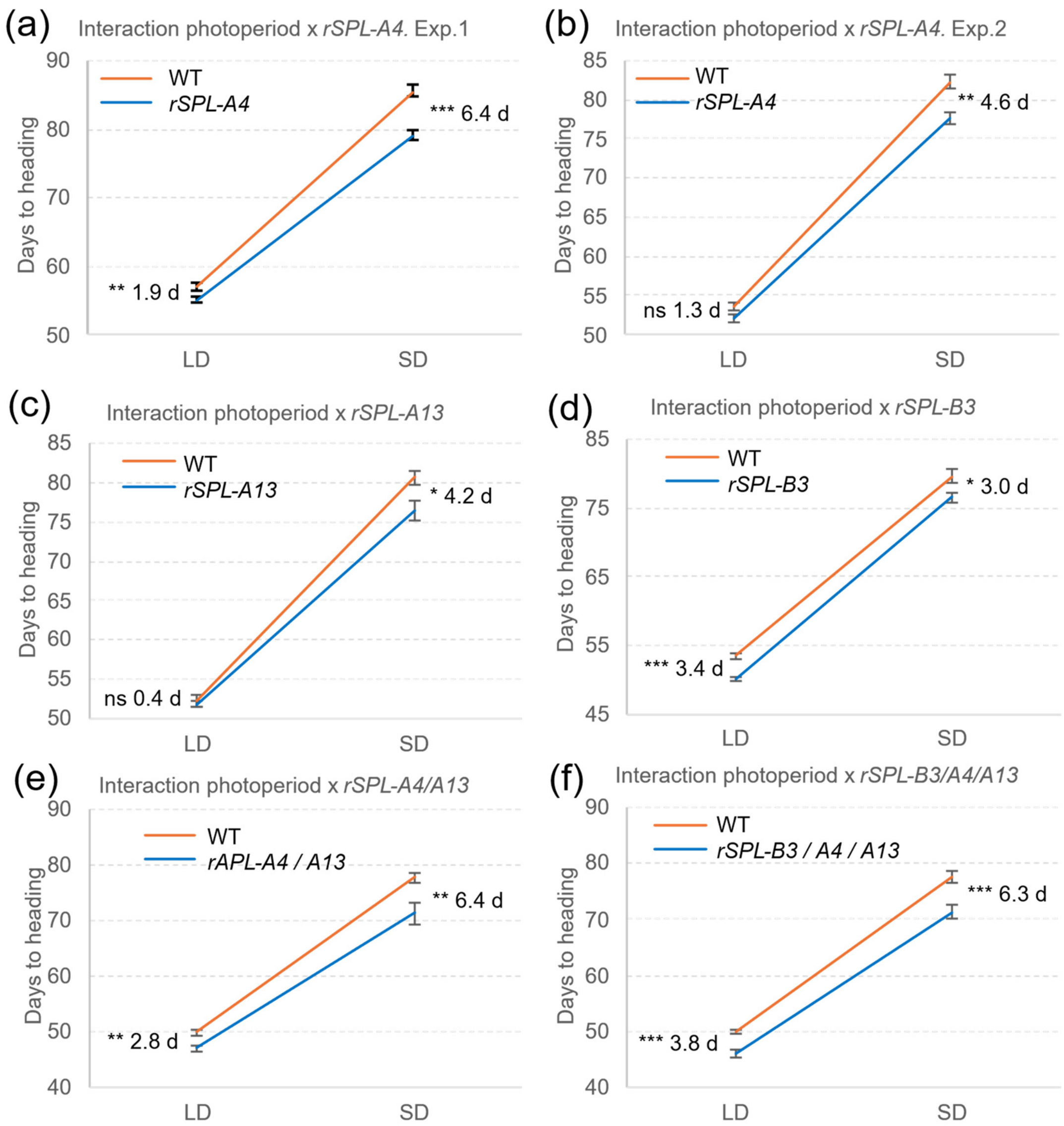

Figure S9. Yeast-one-hybrid assays (Y1H, Method S5).

(**a**) Bait *VRN-A1* promoter (-404 to -350). (**b**) Bait *VRN-B1* intron (+190 +244). (**c**) Bait *FUL-A2* promoter (-836 to -666). (**d**) Bait *FUL-B2* promoter (-992 -826). (**e**) Bait *FUL-A2* intron (+2,556 to +2,652). (**f**) Bait *FUL-B2* intron (+1,347 to +1,514).

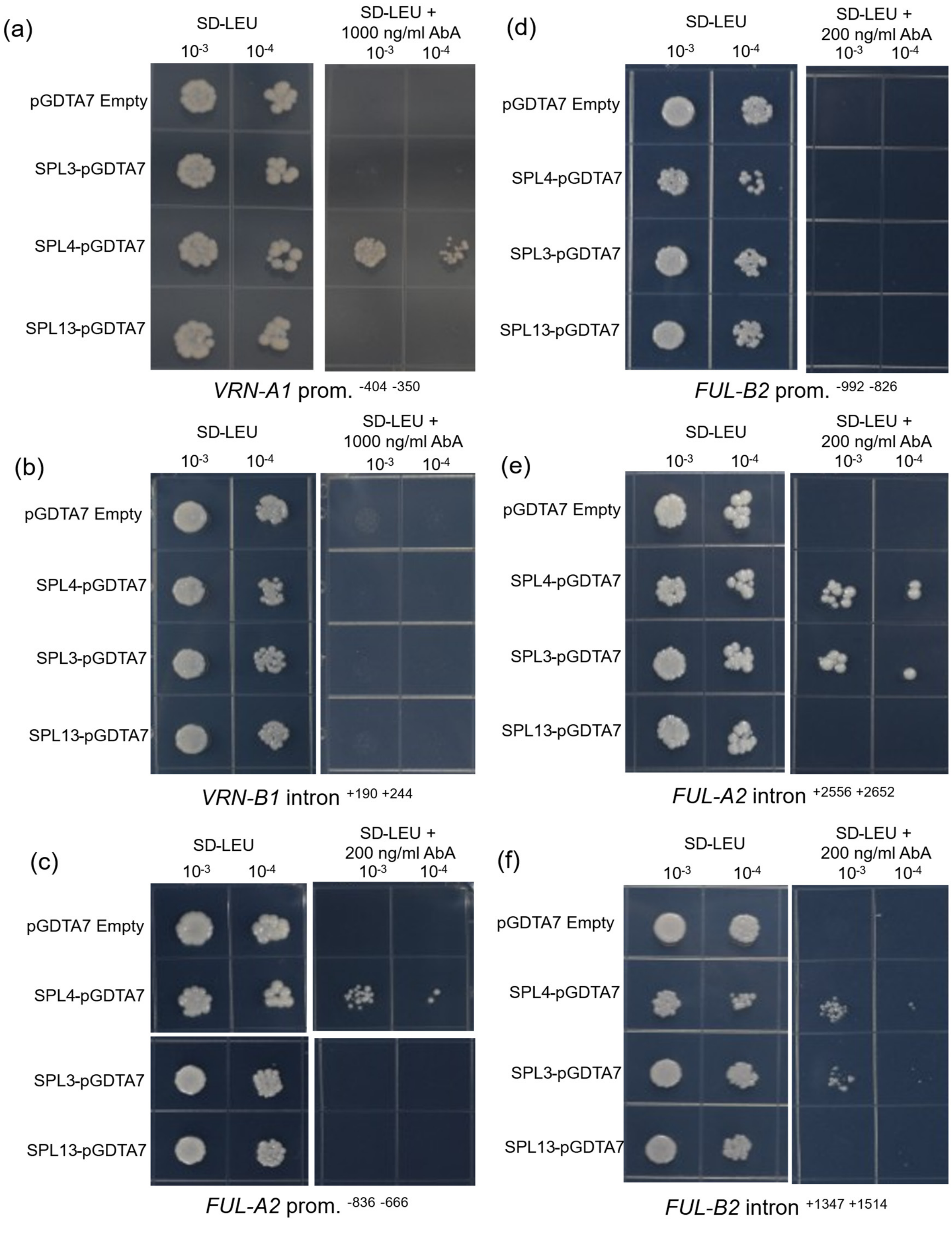

Figure S10. Electrophoretic Mobility Shift Assay (EMSA, Method S6).

(**a**) SDS-PAGE analysis of GST-super1300-SPL fusion protein. M: Marker; 1: GST-SPL (input); 2: GST-SPL (purified). Yellow arrowheads indicate the tagged SPL protein. (**b-d**) Binding of GST-tagged SPL proteins (GST-SPL) to a biotin-labeled DNA probe including a segment of the wheat *FUL-A2* promoter (*FUL-A2*-pr.$^{-399\text{ to }-374}$) with a canonical 'GTACG' SPL-binding site (left panels). A second DNA segment of the *FUL-A2* promoter (*FUL-A2*-pr.$^{-579\text{ to }-551}$) without any *SPL* binding motif was used as a negative control (right panels). Cold probes with the same DNA sequence as the probes but without the biotin label were added at 10x and 50x concentrations to test competition with the labelled probe. The control probe had a random DNA sequence (Data S2) and was included as a second negative control. Black arrows indicate the position of the shifted SPL-DNA complex, with the free probe shown at the bottom. (**b**) SPL3, (**c**) SPL4, (**d**) SPL13.

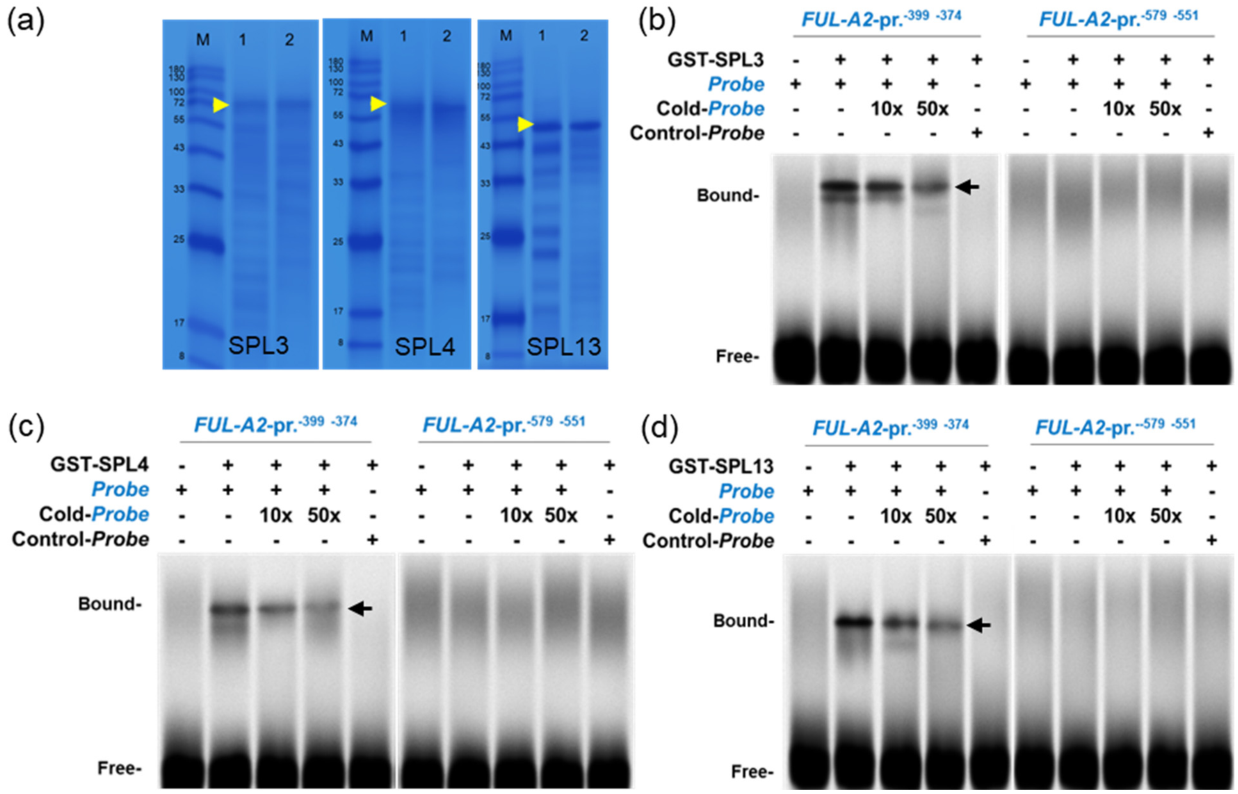

Figure S11. Interactions between DELLA and SPL proteins (Y2H, Method S7).

(a) Yeast-two-hybrid (Y2H) assays showing interactions between a truncated RHT1-GRAS and both SPL3 and SPL4 proteins**. (b)** Y2H assays showing no interactions between a truncated RHT1-GRAS and both SPL13 and a negative control with an empty vector. **(c)** Schematic representation of the three SPL proteins. Green indicates the conserved SBP domain and red the miR156 binding site. **(d)** Schematic representation of two truncated SPL3 proteins lacking 157 or 194 amino acids in the C-terminal region. **(e)** Y2H interactions between DELLA-GRAS and SPL3 truncations Δ157 and Δ194. **(f)** T-COFFEE (v11.0) alignment of partial sequences of wheat SPL3 and SPL4 with Arabidopsis AtSPL2, AtSPL10, and AtSPL11 (these proteins are known to interact with DELLA). The conserved SBP domain and the region critical for the interaction with DELLA are indicated in pink (see color scale below sequence).

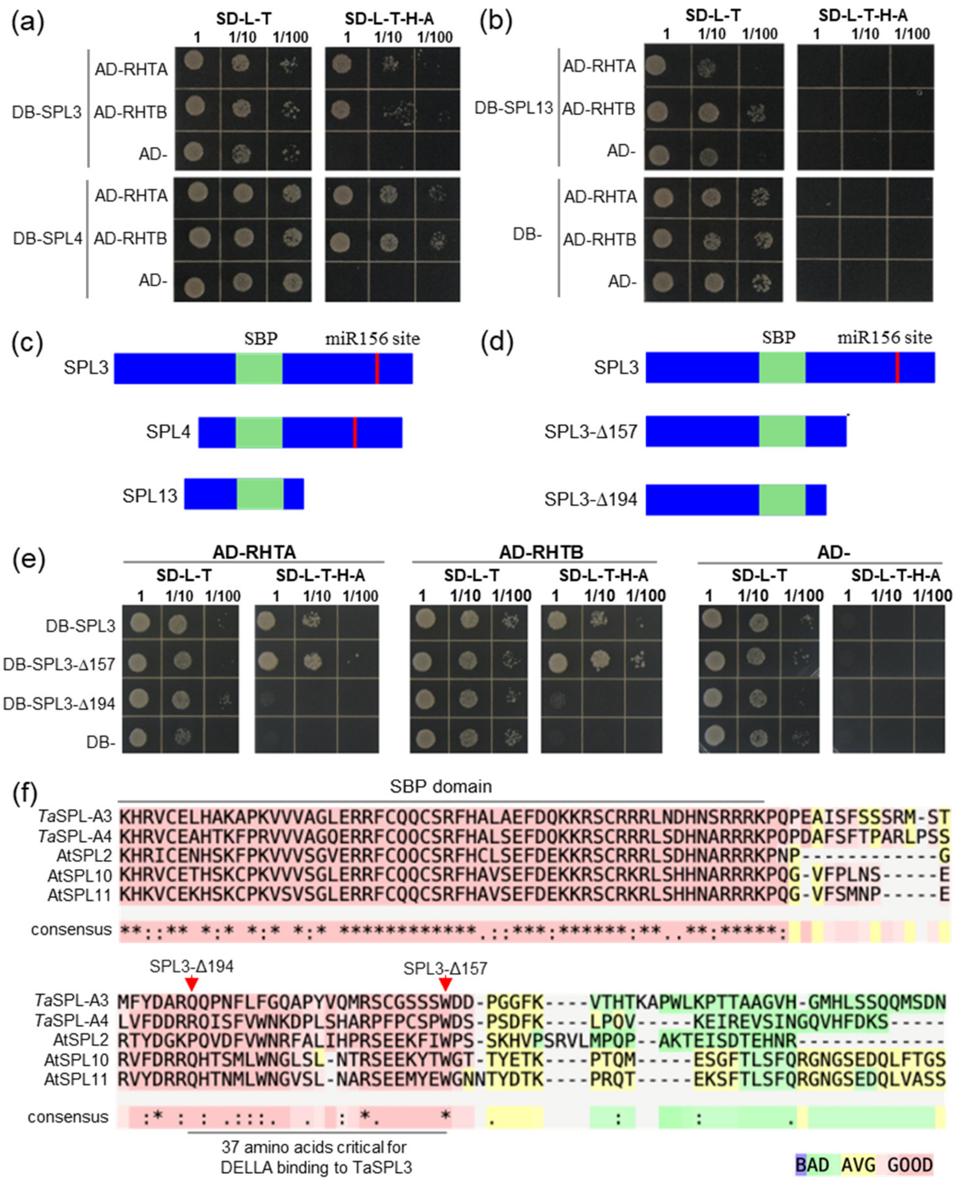

Figure S12. Bi-molecular fluorescent complementation (BiFC or split YFP).

BiFC assays testing the interactions between DELLA-GRASS (= RHT1) and both SPL3 and SPL4 proteins in rice protoplasts. Two independent experimental replications are presented. The three left columns correspond to the 35S::CYFP-RHT1-GRAS vector and the three right columns to the empty CYFP vector (negative control). Each set of three columns includes the YFP, brightfield, and merged pictures. The first two rows are two replications of the co-transformed empty NYFP vector (additional negative control), followed by two replications of the co-transformed 35S::NYFP-SPL3 vector and two replications of the co-transformed 35S::NYFP-SPL4 vector. Bars are 20 µm. No fluorescence signal was detected for the different combinations of empty vectors used as negative controls. See Methods S8.

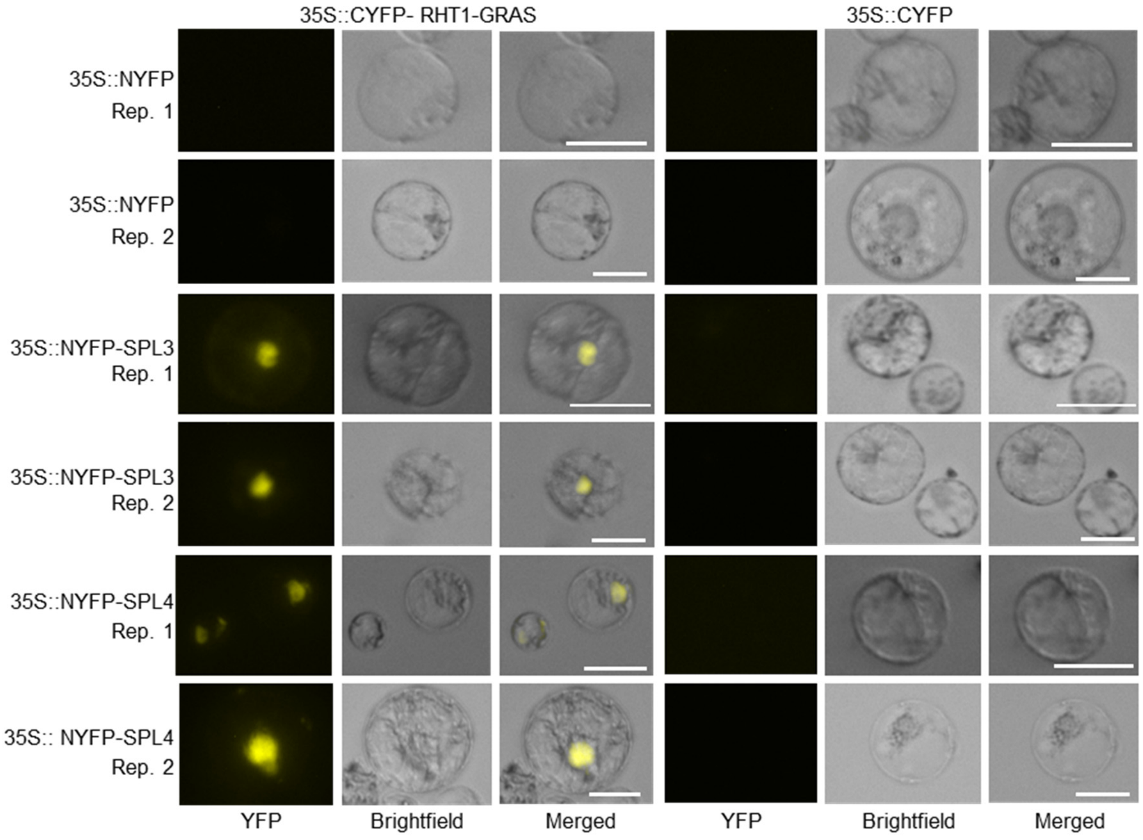

Figure S13. Co-IP experiments showing interactions between DELLA and both SPL3 and SPL4 proteins.

Co-IP assays between DELLA-GRASS (= RHT1) and both SPL3 **(a)** and SPL4 **(b)** proteins in *N. benthamiana*. Seven silent mutations were introduced in the miR156 binding site of *SPL3* and *SPL4* to generate miR156-resistant alleles (see Method S9). Co-IP experiments were performed using GFP-tagged magnetic beads. DELLA-GRASS and the GFP empty vector were detected with anti-GFP antibody, while SPL3 and SPL4 were detected with anti-FLAG antibody. The GFP and FLAG empty vectors served as negative controls.

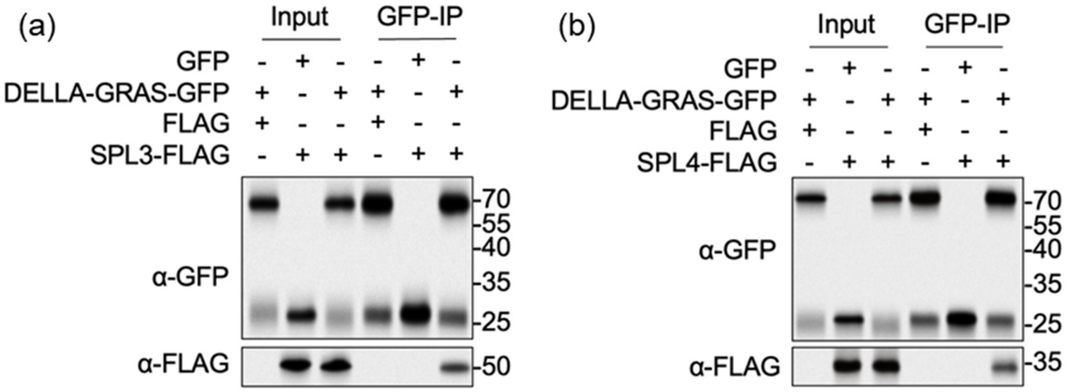

Figure S14. Effects of miR156 and *RHT-B1* alleles on gene expression.

Expression analysis of **(a)** miR156, **(b)** miR172, **(c)** *SPL3*, **(d)** *SPL4*, **(e)** *SPL13* in the third leaves of WT (*Rht-A1a Rht-B1b*, GA-insensitive), MIM156, *Rht-A1a rht-B1*-null (GA-sensitive), and double mutants. Expression was determined by qRT-PCR using *SnoR* as endogenous control for miR156 and miR172 and *ACTIN* for *SPL3*, *SPL4*, and *SPL13*. *P* values are based on Dunnett tests against wild-type (WT). ns= not significant, *= $P< 0.05$, **= $P< 0.01$, ***= $P< 0.001$. Raw data and statistical analyses are available in Data S12.

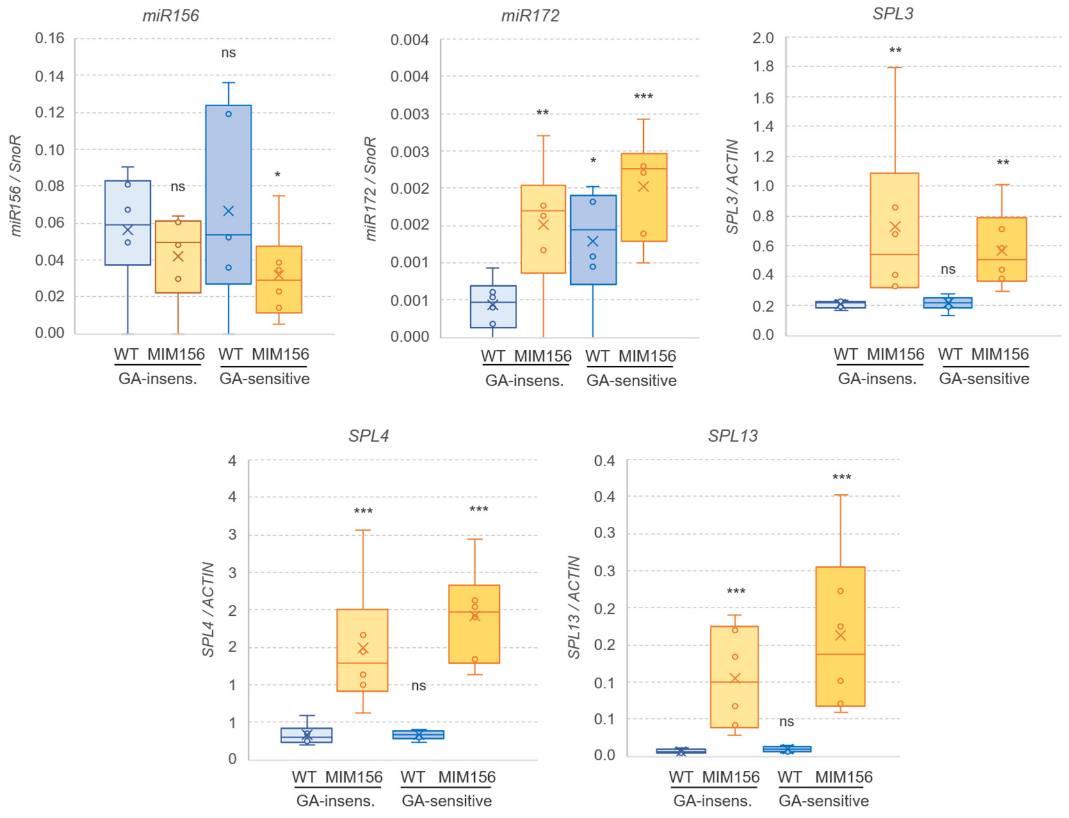

Supplement: Supplementary file 1 — Figure S1. miR156 binding sites in nine wheat SPL genes and their expression profiles. Figure S2. Alignment of SBP domains for Arabidopsis, rice, and wheat SPL proteins. Figure S3. Phylogenetic tree of SPL proteins based on the SBP domain alignment. Figure S4. Regulation of SPL3, SPL4, SPL13, and SPL2 expression by miR156. Figure S5. Expression of wild‐type and resistant alleles rSPL3, rSPL4, and rSPL13. Figure S6. Pictures of wild‐type, rSPL3, rSPL4, and rSPL13 mutants at heading time. Figure S7. Effect of rSPL3, rSPL4, rSPL13 on leaf number. Figure S8. Effect of rSPL3, rSPL4, rSPL13 on heading time under LD and SD. Figure S9. Yeast‐one‐hybrid (Y1H) assays. Figure S10. Electrophoretic mobility shift assay (EMSA). Figure S11. Interactions between DELLA and SPL proteins. Figure S12. Bimolecular fluorescent complementation (BiFC or split YFP). Figure S13. Co‐IP experiments showing interactions between DELLA, and both SPL3 and SPL4 proteins. Figure S14. Effects of miR156 and RHT‐B1 alleles on gene expression. [file TPJ-125-0-s002.pdf]
